# Cancer Research

**Published:** 1910-08-27

**Authors:** 


### Cancer Research.

Although space has already frequently been afforded in these pages to the progress of cancer research and the results hitherto obtained and recorded, no apology is needed for drawing the attention of the profession to the ninth report from the Cancer Research Laboratories of the Middlesex Hospital, recently published. Once more, as so often before, it has to be clearly recognised that the solution of the problem is not yet in sight; but at least there is a constant dissipation of our ignorance which compels us to realise how little we know and how gigantic the subject is. In view of popular impatience, Dr. E. M. Woodman's report on Certain Empirical Modes of Treatment tried during 1909 is noteworthy. The lay mind is apt to complain that doctors are too scientific and not sufficiently practical (a distinction which should import no difference whatever), and are unwilling to try methods of treatment other than those based on complicated theories. Considering the hundreds of empirical cancer cures which have been vaunted since the dawn of history, the profession is well advised to view each successive new one with caution, if not with actual distrust; and a study of the report in question shows how disappointing even the most boldly advertised of these remedies are. Thus, seven cases were treated with aryl-arsonates, and in every case treatment was pushed until symptoms of arsenical poisoning were being manifested; in not one single case was any beneficial result manifested, not even relief of pain. Three cases were treated with the long discredited neoformans vaccine: no result followed beyond a recurrent jaundice in one patient. Coley's fluid was used in three cases of sarcoma and one of carcinoma. In the latter there was slight relief of pain; in two of the former there was no beneficial action. In the third of the sarcoma cases, however, there was a remarkable sequence of events. The patient was a healthy-looking girl of twenty, admitted for a large primary sarcoma of the inguinal glands. She received twenty-eight injections of Coley's fluid, beginning with $\frac{1}{4}$ minim, and increasing to a maximum of 25 minims. These injections produced recurring febrile attacks, which resulted in a progressive anæmia; the treatment was suspended ultimately because this result of it threatened to bring about a fatal issue. On the other hand, the effect on the tumour was remarkable: within a fortnight it began to shrink, and at the end of a month it was estimated to consist of but a fourteenth of its previous bulk; and the skin over it was white and supple, instead of reddened and adherent. Neighbouring enlarged glands became mobile and discrete. Unfortunately, the injections, at first painless, became very painful, and were often followed by local suppuration; at the patient's request, and in view of the severe toxæmia caused, the treatment was given up, the growth speedily increased, and death took place three months later. Finally, pitchblende was used in fourteen cases. The report states that, clinically, pitchblende diminishes the concomitant acute inflammatory reaction, and probably encourages fibrosis in the tumour.. There are many other communications in this publication which are of interest, and perhaps of higher scientific value than those from which quotation has been made; but at least there is no excuse for anyone who claims to have discovered a cancer remedy to say that it is not given a full and fair trial by the experts to whom such duties are informally delegated by the profession at large.

